# Prioritizing cancer-related microRNAs by integrating microRNA and mRNA datasets

**DOI:** 10.1038/srep35350

**Published:** 2016-10-13

**Authors:** Daeyong Jin, Hyunju Lee

**Affiliations:** 1 School of Electrical Engineering and Computer Science, Gwangju Institute of Science and Technology, Gwangju, 500-712, Korea

## Abstract

MicroRNAs (miRNAs) are small non-coding RNAs regulating the expression of target genes, and they are involved in cancer initiation and progression. Even though many cancer-related miRNAs were identified, their functional impact may vary, depending on their effects on the regulation of other miRNAs and genes. In this study, we propose a novel method for the prioritization of candidate cancer-related miRNAs that may affect the expression of other miRNAs and genes across the entire biological network. For this, we propose three important features: the average expression of a miRNA in multiple cancer samples, the average of the absolute correlation values between the expression of a miRNA and expression of all genes, and the number of predicted miRNA target genes. These three features were integrated using order statistics. By applying the proposed approach to four cancer types, glioblastoma, ovarian cancer, prostate cancer, and breast cancer, we prioritized candidate cancer-related miRNAs and determined their functional roles in cancer-related pathways. The proposed approach can be used to identify miRNAs that play crucial roles in driving cancer development, and the elucidation of novel potential therapeutic targets for cancer treatment.

MicroRNAs (miRNAs) are small non-coding RNAs that regulate the expression of target genes by binding to their 3′ untranslated regions. Recent studies aimed at the identification of cancer-related miRNAs revealed that miRNAs significantly affect cancer development by regulating the expression of oncogenes, tumor suppressors, and a large number of other genes, which results in the perturbation of biological networks^1^,^2^. Many computational approaches have been developed for the systemic identification of cancer-related miRNAs and their target genes and elucidation of the functional roles of miRNAs in cancer. These approaches can be broadly summarized into five categories.

First, several algorithms predict miRNA target genes based on the sequence complementary between these genes and miRNAs in the seed regions, and the predicted gene-miRNA interactions can be accessed through databases such as microCosm^3^, Pictar^4^, and TargetScans^5^. However, these predictions, based on sequences alone, cannot explain miRNA mechanisms in cancer development and progression, unless various biological activities, including miRNA-regulated gene and protein expression changes, are not considered.

Additionally, several computational approaches for the prediction of novel miRNA-disease relationships based on the existing biological databases, such as those containing information about miRNA similarities, disease similarities, and experimentally validated miRNA-disease relationships, have been proposed. Xuan *et al*.^6^ assumed that miRNAs related to similar diseases are functionally related. Therefore, they calculated functional similarities between miRNAs based on previously known miRNA-disease relationships, and for each miRNA, they obtained k most similar miRNAs. These similarities were used to infer new miRNAs related to a given disease. Chen *et al*.^7^ formulated miRNA-disease relationship prediction problem as an optimization problem based on regularized least squares using the same assumption as Xuan *et al*.^6^, and demonstrated that the proposed approach successfully recovered miRNAs previously known to be related to several cancer types. In addition to this, they constructed miRNA-disease networks by employing Gaussian interaction profile kernel similarity^8^,^9^ and restricted Boltzmann machines^10^. Pasquier *et al*.^11^ used a vector space model to predict miRNA-disease relationships. They first combined miRNA-disease, miRNA-neighbor, miRNA-gene, miRNA-word, and miRNA-family relationships. Afterward, singular value decomposition was applied for dimension reduction, and miRNAs and disease were represented as vectors, and miRNAs related to diseases were prioritized based on vector similarities. Although these approaches may uncover novel miRNA-disease relationships, they are highly dependent on the previously obtained knowledge, while predicting miRNAs with unknown relationships to any disease is difficult.

Next, several studies used miRNA expression changes to identify cancer-related miRNAs. Iorio *et al*.^12^ showed that the differential expression of miRNAs in ovarian cancer can distinguish cancer cells from normal cells, and that over- and under-expression of miRNAs are associated with pathologic properties, such as histotype and lymphovascular invasion. Srinivasan *et al*.^13^ identified ten signature miRNAs in glioblastoma (GBM) by analyzing miRNA expression data using COX regression analysis, and classifying patients into a low-risk and a high-risk group, based on the survival time. Zhang *et al*.^14^ identified seven differentially expressed miRNAs (DE miRNAs), with their expression significantly associated with the survival time in hepatocellular carcinoma. They associated these seven signature miRNAs with several clinical parameters, such as tumor stage, tumor status, and gender, and found independent prognostic parameters based on univariate and multivariate analysis. DE miRNAs were identified, and survival analysis was performed in these studies in order to identify cancer-related miRNAs. However, some of these miRNAs may not be identified using this approach, due to several reasons, including the use of different processes for the filtering of clinical data, and different cohort sizes^15^. Additionally, miRNAs perform their functions in combination with transcription factors and other genes, and these combinatorial effects, and not miRNA expression alone, may be related to the survival^16^.

Furthermore, computational approaches incorporating negative correlations between gene and miRNA expression levels have accelerated the identification of cancer-related miRNAs. MiRNA-gene pairs have been predicted based on various models, including linear regression, lasso regression, and Bayesian model, and these models have been applied to several cancer datasets^17^. The recent availability of paired miRNA and gene expression levels in multiple cancer datasets found in The Cancer Genome Atlas (TCGA)^18^,^19^,^20^,^21^ allowed simultaneous analysis of miRNA and gene expression in multiple cancer types^22^.

Finally, module-based approaches have been recently proposed, and here modules that contain a set of genes and miRNAs that are highly correlated and involved in the same pathways are identified by integrating multiple types of genomic data, such as gene and miRNA expression levels, gene-gene interactions, and gene-miRNA interactions^23^,^24^,^25^. These studies have highlighted the complex interactions between genes and miRNAs that contribute to the cancer development. Zhang *et al*.^23^ employed a non-negative matrix factorization framework, where miRNA and gene expressions were factorized into a common basis matrix, and gene-miRNA regulatory modules were constructed. They showed that genes and miRNAs in these modules significantly overlaps with previously known cancer-related genes, miRNAs, and biological pathways. Zhang *et al*.^24^ constructed a gene-miRNA network using negative correlations between gene and miRNA expressions and gene-miRNA interactions, and candidate cancer-related miRNAs were prioritized. Using our previous approach^25^, we constructed gene-miRNA modules using a biclustering algorithm and a Gaussian Bayesian network framework. However, these approaches only consider local gene expression changes depending on miRNA expressions, but they do not consider the effects on the whole biological network.

Although significant efforts have been undertaken to identify cancer-related miRNAs, only a small number of studies prioritizes cancer-related miRNAs based on gene and miRNA expressions. For mRNAs, several algorithms have been developed in order to prioritize disease-related genes^26^,^27^, because the functional effects of genes may differ. Similarly, the extents of functional effects of miRNAs in cancer may vary depending on how miRNA expression changes are propagated through the biological network.

Here, we aimed to prioritize miRNAs that lead to significant changes in the whole biological network during cancer initiation and development. We propose a novel approach based on order statistics that prioritizes miRNAs whose expression changes significantly affect cancer development. Additionally, we explain functional roles of the miRNAs highly ranked in our model at the pathway level.

## Methods

**Figure 1:**
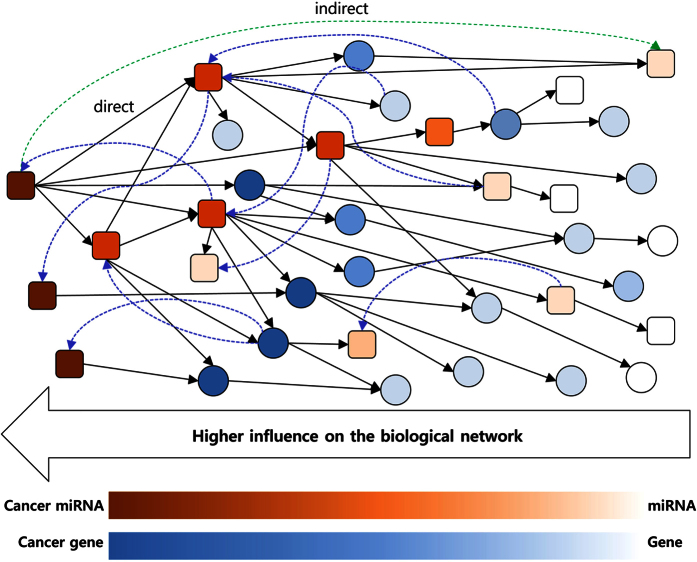
Biological network structure. Cancer-related miRNAs (dark orange rectangles) and genes (dark blue circles) affect other genes and miRNAs and ultimately the entire biological network. In contrast to this, white rectangles and circles represent miRNAs and genes that do not affect other genes and these miRNAs are mostly regulated by the previous category of genes and miRNAs. Black solid lines and green dotted lines represent the direct or indirect changes, respectively, of one node that affects linked nodes. Although we presented only one indirect edge for simplicity, there are many indirect relationships in a real biological network. Right nodes influence on the left nodes shown in blue dotted lines, however, influences from left to right are more abundant globally.

**Figure 2:**
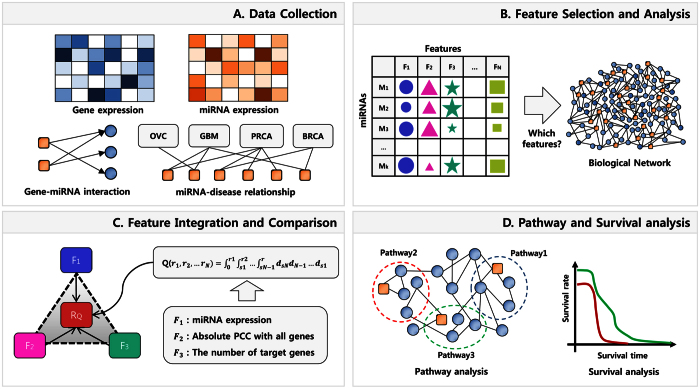
Schematic overview of the proposed approach. (**A**) Gene and miRNA expressions in paired samples, gene-miRNA interactions, and miRNA-disease relationships are collected. (**B**) Selection and analysis of cancer-related miRNA features. (**C**) Integration of features F1, F2, and F3 selected in the step (**B**) and calculation of integrated rankings *R*_*Q*_ using order statistic. (**D**) Pathway and survival analysis for the understanding of functional roles of miRNAs in biological pathways.

As shown in Fig. 1, we assumed that some genes and miRNAs, illustrated in the left part of the presented biological network, have many interactions and cause significant alterations of other genes/miRNAs, directly or indirectly. Therefore, they are more likely to contribute to the development of cancer than genes and miRNAs represented in the right part of the network, which are mainly affected by the previous category of genes and miRNAs, and are less likely to contribute to the cancer development. A schematic overview of our approach is presented in Fig. 2, while the details are described below.

### Data collection

We obtained microarray and/or RNA-Seq datasets for GBM, ovarian cancer (OVC), prostate cancer (PRCA), and breast cancer (BRCA) from the TCGA data portal (http://cancergenome.nih.gov). Paired datasets of gene and miRNA expressions were obtained.

For microarray, gene and miRNA expression data were generated using Affymetrix HG-U133A and Agilent H-miRNA_8 × 15 for GBM and OVC, respectively. GBM dataset contains 12,042 genes and 470 mature miRNAs, obtained from 482 tumor samples, and OVC dataset contains 12,042 genes and 723 mature miRNAs obtained from 578 tumor samples. For RNA-Seq, gene and miRNA expression datasets were generated by IlluminaHiSeq_RNASeqV2 and IlluminaHiSeq_miRNASeq, respectively, using OVC, PRCA and BRCA samples. OVC dataset contains 20,806 genes from 416 tumor samples, PRCA dataset contains 20,531 genes from 494 tumor samples, and BRCA dataset contains 20,532 genes from 461 tumor samples. Additionally, they commonly contain 1,046 miRNAs obtained from the paired samples with genes.

Predicted gene-miRNA interactions were collected from microCosms^3^, PicTar^4^, and TargetScans^5^. The information about miRNA-disease relationships was obtained from the Human microRNA Disease Database (HMDD)^28^. We collected OVC miRNA data using “Ovarian Neoplasm” term, GBM miRNAs using “Glioblastoma” or “Glioma” terms, PRCA miRNA data using “Prostatic Neoplasms” term, and BRCA miRNA data using “Breast Neoplasms” term.

### Feature selection and analysis

We propose three miRNA features that may strongly affect the biological network. First, we assumed that highly expressed miRNAs show a high potential to affect the biological network. Therefore, the average miRNA expressions from all cancer samples are selected as the first feature (F1). *F*_*a*_ = {*a*_1_, …, *a*_*i*_, …, *a*_M_}, where *a*_*i*_ is the average expression of a miRNA *i* in the cancer samples, and *M* represents the number of miRNAs. Note that we only considered the expression levels of miRNAs in cancer cells, but not in the normal cells.

Additionally, we assumed that if a miRNA significantly affects some genes, the expressions of this miRNA and the genes may be highly correlated. A miRNA can directly regulate a set of genes, which may indirectly lead to the alterations in the expression of many other genes. Therefore, we considered all genes in the biological network and used the average of absolute Pearson correlation coefficients (PCCs) between miRNA and all gene expressions as the second feature (F2). *F*_*c*_ = {*c*_1_, …, *c*_*i*_,…*c*_*M*_}, where *c*_*i*_ is the average of absolute PCC values between miRNA *i* and all genes in the cancer samples.

We further assumed that miRNAs that bind to many genes strongly affect the biological network. However, only a small fraction of miRNA target genes has been experimentally validated, and therefore, we used computationally predicted gene-miRNA interactions, based on sequence complementary. We obtained the predicted gene-miRNA interactions from microCosms^3^, PicTar^4^, and TargetScans^5^. All interaction pairs were extracted from these three databases and duplicated interaction pairs were removed. Furthermore, we counted the number of the predicted targets for each miRNA and these numbers can be considered the numbers of potential interacting genes, representing our third feature (F3). *F*_*t*_ = {*t*_1_,…, *t*_*i*_,…, *t*_*M*_}, where *t*_*i*_ is the number of target genes for miRNA *i*. Note that, because the number of predicted targets is determined by the sequence complementary information, gene-miRNA interactions for the third feature are the same, regardless of the cancer type used.

### Integration of features

**Figure 3:**
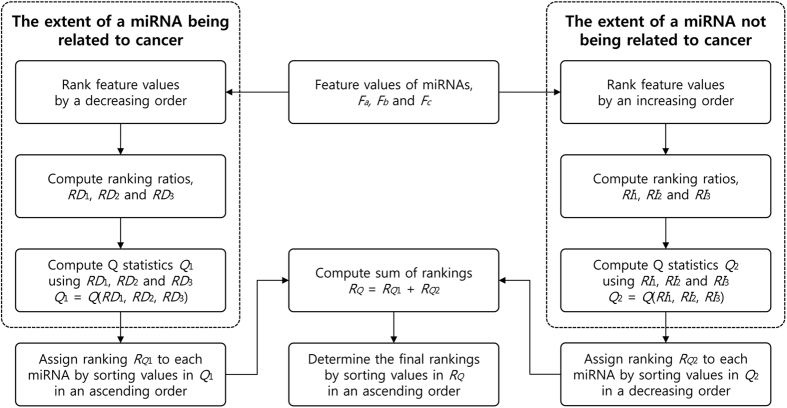
Flowchart showing the feature integration process. We computed feature values for each miRNA. Following this, we computed ranking ratios by decreasing (*RD*) and increasing order (*RI*). Afterward, we computed Q statistics, *Q*_1_ and *Q*_2_, and rankings, *R*_*Q*1_ and *R*_*Q*2_. Finally, we determined the final rankings by the addition of these two rankings.

We used order statistics to integrate the three features, F1, F2, and F3. In Fig. 3, a flowchart of feature integration process is presented. First, we computed the ranking ratios for the values of each feature. *F*_*a*_, *F*_*c*_, and *F*_*t*_ values were ranked in a decreasing order and their ranking ratios were stored in *RD*_1_, *RD*_2_, and *RD*_3_, respectively. Let *RD*_1_ = 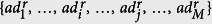, where 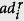 is the ranking ratio of *a*_*i*_ ∈ *F*_*a*_ and 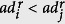 if *a*_*i*_ > *a*_*j*_. Similarly, *RD*_2_ = 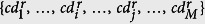, where 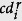 is the ranking ratio of *c*_*i*_ ∈ *F*_*c*_ and 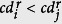 if *c*_*i*_ > *c*_*j*_, and *RD*_3_ = 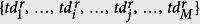, where 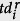 is the ranking ratio of *t*_*i*_ ∈ *F*_*t*_ and 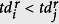 if *t*_*i*_ > *t*_*j*_. Furthermore, for a given miRNA *i*, a Q statistic is computed based on joint cumulative distribution of order statistic of the three features^26^. The integration was approximated using a recursive solution proposed by Stuart *et al*.^29^.


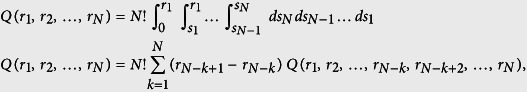


where *N* = 3, the number of the features, *r*_0_ = 0, and *r*_*k*_ is the ranking ratio for the *k*th feature for the given miRNA *i*: 
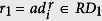
, 
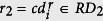
, and 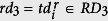. For all miRNAs, Q statistics were calculated, so that *Q*_1_ = {*qd*_1_,…, *qdi*, …, *qd*_*M*_}, where *qd*_*i*_ is the Q statistic for a miRNA *i*. More specifically, 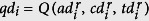.

We assigned a ranking to each miRNA by sorting values in *Q*_1_ in an ascending order. Let *R*_*Q*1_ = 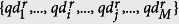, where 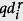 is a ranking of *qd*_*i*_ ∈ *Q*_1_ and 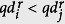 if *qd*_*i*_ < *qd*_*j*_. We assumed that a miRNA *i* with a smaller value of *qd*_*i*_ (thus, with a higher ranking) is more highly related to cancer development, since we assumed that miRNAs with larger *F*_*a*_, *F*_*c*_, and *F*_*t*_ values are more likely to be related to cancer, resulting in smaller *qd*_*i*_ values. Therefore, *R*_*Q*1_ is a set of miRNA rankings, indicating their relevance to cancer development. However, some miRNAs are highly ranked in some features but not in others. In these cases, miRNA rankings can be dominantly determined by a single or a small number of features because the ranking in *R*_*Q*1_ becomes higher with a lower number of high ranking features. To reduce the effects produced by these few features, we incorporated the following step.

We calculated another Q statistic that measures the extent to which a miRNA is not related to cancer. When *F*_*a*_, *F*_*c*_, and *F*_*t*_ feature values are small, it is unlikely that a miRNA is related to cancer. Hence, *F*_*a*_, *F*_*c*_, and *F*_*t*_ values are ranked in an ascending order and their ranking ratios are stored in *RI*_1_, *RI*_2_, and *RI*_3_, respectively. Let *RI*_1_ = 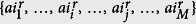, where 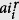 is the ranking ratio of *a*_*i*_ ∈ *F*_*a*_ and 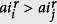 if *a*_*i*_ > *a*. Similarly, *RI*_2_ = 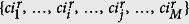, where 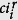 is the ranking ratio of *c*_*i*_ ∈ *Fc* and 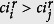 if *c*_*i*_ > *c*_*j*_, and *RI*_3_ = 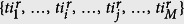, where 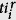 is the ranking ratio of *t*_*i*_ ∈ *F*_*t*_ and 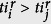 if *t*_*i*_ > *t*_*j*_. Afterward, rankings in *RI*_1_, *RI*_2_, and *RI*_3_ are integrated using Q statistics. Let *Q*_2_ = {*qi*_1_, …, *qi*_*i*_, …, *qi*_*M*_}, where *qi*_*i*_ is the Q statistic for a miRNA *i* obtained by integrating its rankings in *RI*_1_, *RI*_2_, and *RI*_3_. More specifically, 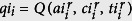. Because *Q*_2_ measures the extent of a miRNA not being related to cancer, we sorted them in a decreasing order and stored the rankings into *R*_*Q*2_. Let *R*_*Q*2_ = 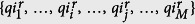, where 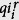 is a ranking of *qi*_*i*_ ∈ *Q*_2_ and 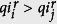 if *qi*_*i*_ < *q*_*ij*_. *R*_*Q*2_ represents a set of miRNA rankings, indicating their relevance to cancer development.

Final rankings were determined using a sum of these two rankings: *R*_*Q*_ = *R*_*Q*1_ + *R*_*Q*2_. We used *R*_*Q*_ as a new statistic to determine all miRNA rankings, penalizing miRNA rankings that did not show significant feature values for some of the three examined features. Several examples of integrating features are described in Supplementary Table 1.

### Pathway analysis related to miRNA

In order to understand the functional roles of miRNAs in biological pathways, we performed a pathway enrichment analysis using KEGG pathways and gene ontology (GO) biological functions. We downloaded 186 KEGG pathways and 825 GO biological functions from Gene Set Enrichment Analysis (GSEA, http://www.broadinstitute.org/gsea), and extracted experimentally validated gene-miRNA interaction datasets from miRTarbase^30^. Strong evidence, such as reporter assays, western blotting, and qPCR and/or weak evidence, such as microarrays, next generation sequencing (NGS), and pSILAC support these datasets. For each miRNA, we selected genes with expression levels highly correlated with the given miRNA expression levels (1% of genes in PCC values) or those interacting with that miRNA according to miRTarbase^30^. Finally, we performed a pathway enrichment analysis using a hypergeometric test and obtained *p*-values for each miRNA. *p*-values were corrected to *q*-values based on Benjamini & Hochberg correction^31^, in order to address multiple comparison issues. We considered pathways with *q*-value less than 0.05 as significant.

### Survival analysis

We performed a survival analysis to identify miRNAs that play crucial roles in cancer patient survival. Clinical information about GBM and OVC samples was obtained from TCGA. For each candidate miRNA, we divided these samples into two groups: an under-expressed group, where the expression levels of that miRNA belong to the bottom 10% of all values, and an over-expressed group, where the expression levels of the miRNA belong to the top 10% of values. We performed Kaplan-Meier survival analysis and obtained *p*-values.

## Results

### Feature selection and analysis

**Figure 4:**
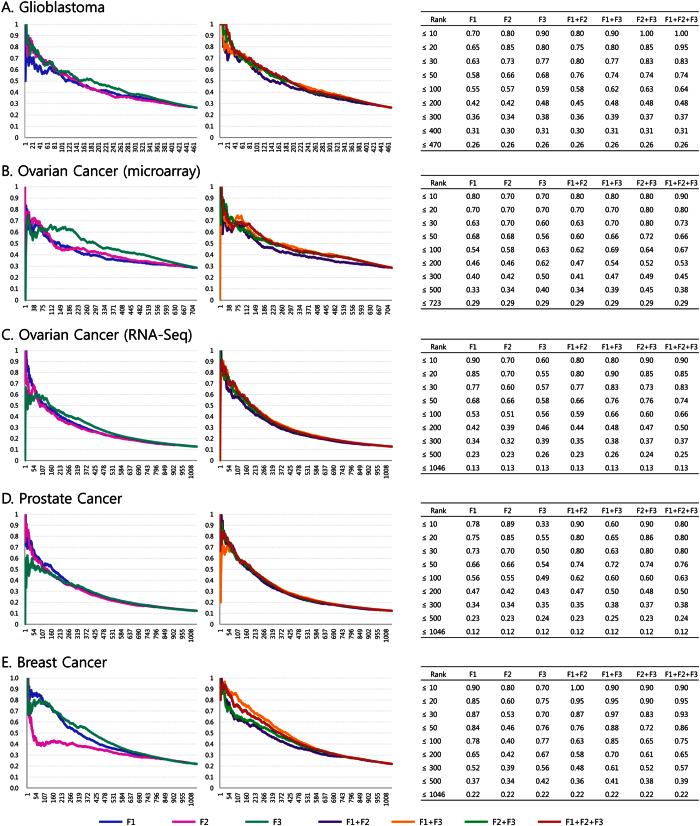
Performances of features selected for the ranking of cancer-related miRNAs. *x*-axis represents miRNA rankings and *y*-axis represents a cumulative ratio of cancer-related miRNAs included in HMDD. (**A–E**) correspond to GBM, OVC (microarray), OVC (RNA-Seq), PRCA and BRCA samples, respectively. Left, cumulative ratios of cancer-related miRNAs are presented, which were obtained using a single feature. Right, cumulative ratios of cancer-related miRNAs, by integrating features, are presented. For most cancer types, cumulative ratios of cancer-related miRNAs increase with the rank. Additionally, the integration of more than one feature shows higher performance than when only one feature is used. Furthermore, integration of all features shows the highest performance for most cancer types.

For each miRNA, we computed three feature values, F1, F2, and F3. To show that these three features are cancer-related, we ranked all miRNAs based on the feature values. We assigned a value of one to miRNAs if they were found in the HMDD database and zero if not. We computed a cumulative ratio by computing the average number of miRNAs that had the value of one for each ranking. Afterward, we determined whether the higher-ranked miRNAs have higher cumulative ratios. In Fig. 4, cumulative ratios of cancer-related miRNAs for the five cancer datasets are presented. Images on the left in Fig. 4 show that cumulative ratios of all features, F1, F2, and F3, have a similar tendency of steady decrease with the ranking, suggesting that highly ranked miRNAs are more likely to be related to cancer regardless of cancer type (GBM, OVC, PRCA, and BRCA) and platforms (microarray or RNA-Seq).

### Integration of features

We integrated the three features (F1, F2, and F3) using order statistics and ranked miRNAs based on *R*_*Q*_. As shown in Fig. 4, the cumulative ratios of cancer-related miRNAs were shown to be the highest when these three features were integrated, compared with single-feature or two-feature analyses. Additionally, we applied different integration methods: an average of ranking ratios of the three features, F1, F2, and F3 (e.g. (*RD*_1_ + *RD*_2_ + *RD*_3_)/3), an inverse normal transformation^32^, and two other order statistics, *R*_*Q*1_ and *R*_*Q*2_. The comparison of the proposed integration method (*R*_*Q*_) with the other tested methods showed that our method had the highest performance on average when GBM, OVC (microarray), OVC (RNA-Seq), PRCA, and BRCA samples were used (Supplementary Table 2). These results suggest that the integration of these features assists the identification of cancer-related miRNAs. Supplementary Tables 3, 4, 5, 6, and 7 show miRNA rankings, integrated scores and cumulative ratios of cancer-related miRNAs for GBM, OVC (microarray), OVC (RNA-Seq), PRCA, and BRCA samples, respectively.

#### GBM-related miRNAs

Of 470 miRNAs found in the GBM dataset, 123 miRNAs (26.3%) were shown to be related to GBM according to HMDD. In our analysis, 19 out of the top 20 miRNAs (95.0%) and 64 out of the top 100 miRNAs (64.0%) were shown to be related to GBM (Fig. 4A), which demonstrates a significant overlap with GBM-related miRNAs (*p*-value = 4.43e–20, in the hypergeometric test).

Furthermore, we investigated whether 98 previously known GBM-related genes^33^,^34^ interact with miRNAs ranked high in our analysis. The target genes of the top 20 ranked miRNAs obtained from miRTarbase^30^ included 3949 genes and 515 genes, found to interact with at least one of the miRNAs with some evidence and with strong evidence, respectively. Among these, 68 and 43 genes were common with the 98 GBM genes, with some evidence and strong evidence, respectively, showing statistically significant overlaps (*p*-values < 1.12e–13 in the hypergeometric test). Extracted gene-miRNA interactions are listed in Supplementary Table 8.

#### OVC-related miRNAs–microarray dataset

Of 723 miRNAs in OVC microarray dataset, 206 (28.6%) were found in HMDD. In our analysis, 16/20 miRNAs (80%) and 67/100 (67.0%) miRNAs were shown to be OVC-related miRNAs included in HMDD (Fig. 4B), showing a significant overlap (*p*-value = 3.41e-18 in the hypergeometric test).

We further investigated whether previously known 379 OVC genes^33^,^34^ interact with the highly ranked miRNAs. The top 20 ranked miRNAs are known to interact with 4485 genes and 559 genes with some evidence and strong evidence in miRTarbase^30^, respectively. Of these genes, 183 and 89 genes were found among 379 OVC genes with some evidence and strong evidence, respectively, showing statistically significant overlaps (*p*-values < 5.37e-06 in the hypergeometric test). In Supplementary Table 9, a list of gene-miRNA interactions is presented.

#### OVC-related miRNAs–RNA-Seq dataset

In RNA-Seq OVC dataset, among 1046 identified miRNAs, 133 (12.7%) are included in HMDD as OVC-related. Our analyses showed that 17/20 miRNAs (85%) and 66/100 (66.0%) are included in HMDD as OVC-related genes (Fig. 4C), demonstrating a significant overlap with OVC-related miRNAs (*p*-value = 8.88e-42 in the hypergeometric test).

#### PRCA and BRCA-related miRNAs

PRCA dataset contained 1046 miRNAs, and 128 (12.2%) are indicated in HMDD as well. In our analysis, 16 out of 20 miRNAs (80.0%) and 63 out of 100 miRNAs (63.0%) were shown to be PRCA-related (Fig. 4D). In the BRCA dataset, 229 (21.9%) miRNAs, of 1046, were found in HMDD as well. Our analysis, integrating two features, F1 and F3, showed the best performance in the identification of BRCA-related miRNAs. However, the integration of all features shows good performance in general. This analysis identified 19 out of 20 miRNAs (95.0%) and 75 out of 100 miRNAs (75.0%) as related to BRCA (Fig. 4E).

### Pathway analysis

We investigated biological pathways affected by cancer-related miRNAs identified in our study. For each miRNA, we calculated PCCs between the expression level of all genes and the given miRNA, and selected highly correlated genes, with PCC values within top 1%. These highly correlated genes and genes that are reported to interact with a specific miRNA in miRTarbase^30^ are considered candidate genes related to the given miRNA.

Furthermore, we constructed reference cancer-related pathways for GBM and OVC. We performed a pathway enrichment test using 98 GBM genes curated from two previous reports^33^,^34^, and 379 OVC genes curated from Dragon Database For Exploration Of Ovarian Cancer Genes (DDOC)^35^ against KEGG pathways. As a result, 61 and 73 pathways were shown to be significantly enriched (*q*-value < 0.05) and used as reference GBM and OVC, respectively, related pathways (Supplementary Tables 10 and 11).

**Figure 5:**
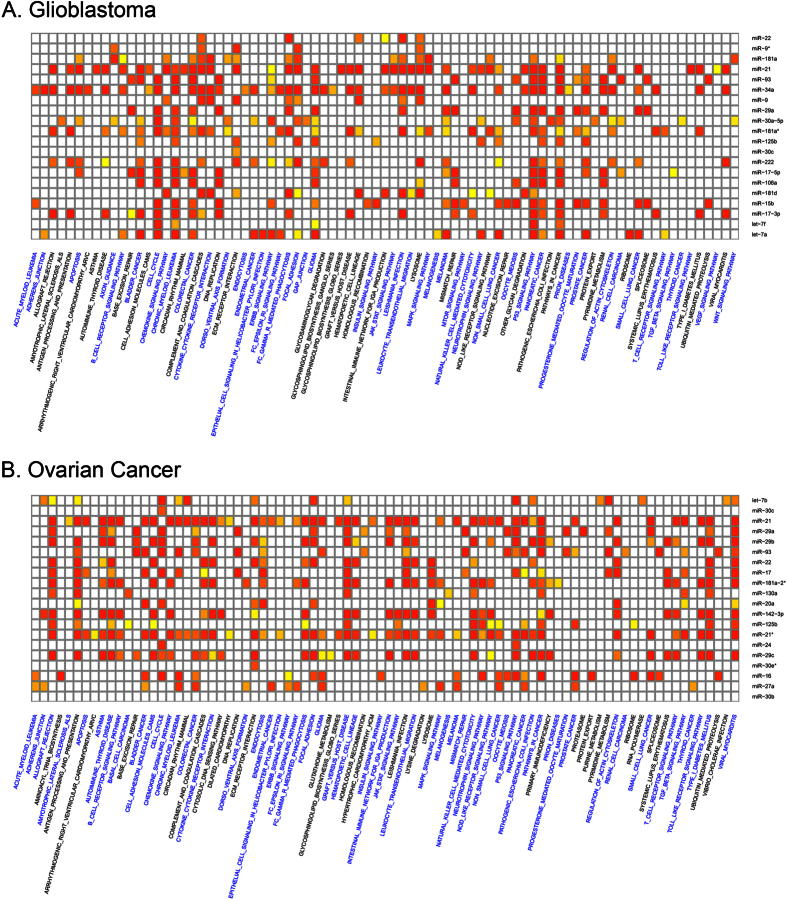
GBM and OVC (microarray) pathway analysis. (**A,B**) Show heatmaps of pathways related to the top 20 miRNAs obtained by analyzing GBM and OVC samples, respectively. Red, orange, yellow, and white colors represent the correlation of a miRNA and the pathway (form high to low, based on *p*-value using a hypergeometric test). Cancer-related pathways are presented in blue.

Pathway enrichment analysis, using the genes related to top 20 GBM candidate miRNAs, is presented in Fig. 5A. A number of pathways (81) were shown to be associated with top 20 miRNAs (Supplementary Table 12). Among them, 47 pathways are reference cancer-related pathways. They include known GBM-related pathways, such as apoptosis^36^, cell cycle^37^, cytokine-cytokine receptor interaction^38^, ErbB signaling pathway^39^, JAK-STAT signaling pathway^40^, MAPK and mTOR signaling pathway^41^, p53 signaling pathway^42^, TGF-beta signaling pathway^43^, VEGF signaling pathway^44^, and Wnt signaling pathway^45^. Additionally, several associations between miRNAs and GBM-related pathways shown in Fig. 5A were previously reported. MiR-181a, miR-21, miR-34a, miR-30a-5p, miR-222, and miR-17-5p were shown to be involved in the apoptosis of glioma cells^46^,^47^,^48^,^49^,^50^,^51^. MiR-21, miR 34a, and miR-30a-5p may be involved in ErbB signaling pathway^52^,^53^,^54^ and miR-21, miR-34a, miR-125b, miR-17-5p/3p, miR-106a, and miR-222 were identified in the p53 signaling pathway in GBM^50^,^51^,^55^,^56^,^57^,^58^. MiR-30a is involved in Wnt signaling pathway through the regulation of PRDM1 during glioma cell growth^59^. Kwak *et al*.^60^ showed that miR-21 is involved in glioma invasion by controlling MAPK signaling pathway.

In OVC analysis, 84 pathways were associated with the top 20 OVC candidate miRNAs (Fig. 5B and Supplementary Table 13). Among them, 56 pathways represent reference cancer-related pathways for OVC, including previously known OVC related pathways, such as apoptosis^61^, ECM receptor interaction^62^, ErbB signaling pathway^63^, JAK-STAT signaling pathway^64^, p53 signaling pathway^65^, and TGF-*β* signaling pathway^66^. Additionally, in Fig. 5B, previously known associations between miRNAs and pathways are presented. MiR-21 and miR-17 were shown to be involved in OVC cell apoptosis^67^,^68^ in p53 signaling pathway in OVC cells^69^,^70^.

Additionally, we performed a functional enrichment test for GO biological functions using the genes related to top 20 GBM and OVC candidate miRNAs. Among 1123 enriched terms for GBM, 290 terms were related to cancer hallmark signatures such as apoptosis, immune response, chromosome abnormalities, inflammation, and angiogenesis^71^,^72^. Similarly, for OVC, 171 out of 791 enriched terms were related to cancer, showing that the top ranked miRNAs significantly affect cancer-related pathways. Enriched terms are listed in Supplementary Tables 14 and 15.

### Survival analysis

**Figure 6:**
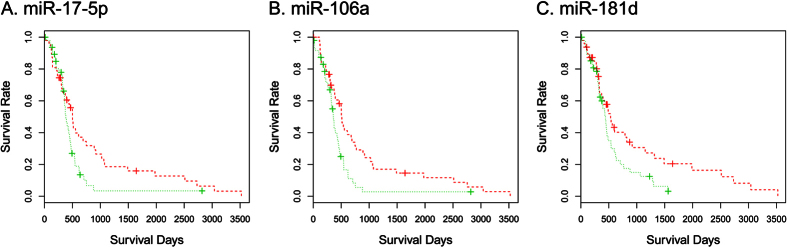
GBM survival analysis. Green and red lines represent samples with underexpressed miRNAs and overexpressed miR-17-5p, miR-106a, or miR-181d, respectively. These miRNAs function as tumor suppressors, and the survival rates of patients with the overexpression of these miRNAs are higher than those of patients with the underexpression of these miRNAs.

We obtained clinical information about 480 GBM and 561 OVC (microarray) samples. Out of 480 GBM patients, 377 were shown to be deceased and the average survival is 493 days. Out of 561 OVC patients, 290 were deceased and the average survival is 1088 days. Considering these data, GBM is more aggressive than OVC. For each miRNA, we divided the samples into 10% underexpressed group and 10% overexpressed group and performed the survival analysis. For GBM samples, 10 out of the top 20 miRNAs (50%) and 29 out of the top 100 miRNAs (29%) were shown to be significantly related to the survival of patients. In Fig. 6, survival curves for samples containing overexpressed or underexpressed miR-17-5p, miR-106a, and miR-181d, which function as tumor suppressors, are shown. Overexpressed groups show longer survival time compared with that of the underexpressed groups. These miRNAs were previously identified as tumor suppressors, as presented in Table 1. For OVC, only six out of the top 100 miRNAs (6%) were shown to be related to the survival of patients. Although multiple studies showed that the highly ranked miRNAs identified in this study are related to OVC, for most of them, no significant difference in the survival time was observed. This may be a result of the higher survival ratio of patients with OVC compared with GBM patients and relatively small differences in the expression levels between the samples. Details of the survival analysis for the top 100 miRNA are described in Supplementary Tables 16 and 17 for GBM and OVC samples, respectively.

### Comparison with other methods

We compared performances of other methods with that of our method using GBM and OVC (microarray) datasets. We used differentially expressed miRNAs for comparison, because the identification of miRNAs based on differential expression between cancer samples and normal samples is the most commonly used approach for the selection of cancer-related miRNAs. We additionally downloaded eight and 10 unmatched normal GBM and OVC (microarray) samples, respectively, computed *p*-values using *t*-test, and ranked miRNAs based on *p*-values.

For comparison, we employed our previous approach^25^ as well, in which gene and miRNA expressions and gene-gene interactions are integrated using a biclustering algorithm and a Gaussian Bayesian network framework, followed by construction of gene-miRNA modules. The inclusion of genes and miRNAs in the same module was explained through the direct regulation of genes by miRNAs and/or indirect regulation by transcription factors. We assumed here that the miRNAs included in the modules are cancer-related. We listed all miRNAs in the modules without duplication and obtained 95 GBM-related and 108 OVC-related candidate miRNAs.

**Figure 7:**
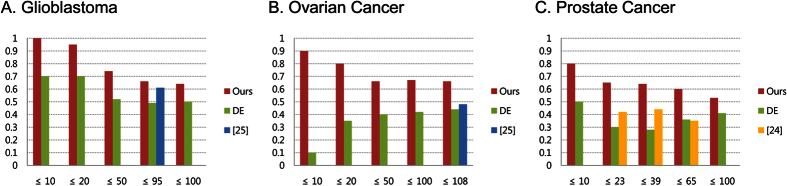
Comparison of our method with other methods. Our approach was compared with the approach based on DE miRNAs, Zhang *et al*.^24^, and our previous approach^25^. Each bar represents a cumulative ratio of cancer-related miRNAs in HMDD^28^ and miRNAs identified by each of the presented methods for each cancer type. The number of miRNAs identified by these methods is represented on *x*-axis. In (**A,B**), GBM and OVC (microarray) datasets from TCGA were used while, in (**C**), PRCA dataset (GSE34933)^24^ from NCBI GEO was used for comparison.

In Fig. 7, each bar represents a cumulative ratio of GBM miRNAs in HMDD (Fig. 7A) and OVC miRNAs in HMDD (Fig. 7B) among miRNAs identified by each method, and on the *x*-axis are represented the numbers of candidate miRNAs selected based on the rankings in each method. Across all rankings ranging from 10 to 100, our method outperformed the other two investigated approaches. Although our method showed a slightly higher performance when 95 GBM candidate miRNAs were used, in comparison with our previous approach (66.3% vs. 61.1%, respectively), it significantly outperformed our previous approach when using 108 OVC candidate miRNAs (66.3% vs 48.1%, respectively).

An approach proposed by Zhang *et al*.^24^ was also employed for comparison. Zhang *et al*.^24^ identified differentially expressed genes and miRNAs, computed PCCs between them, and constructed a gene-miRNA network by intersecting negative PCC pairs and combination of experimentally and computationally derived gene-miRNA interactions. They ranked candidate cancer-related miRNAs by considering the number of genes uniquely regulated by miRNAs in the obtained network. When the method was applied to a GSE34933 dataset from NCBI GEO, containing the information about gene and miRNA expression for four PRCA samples and benign prostatic hyperplasia samples, 26, 39 and 69 PRCA candidate miRNAs were identified depending on the thresholds. In Fig. 7C, it is clearly demonstrated that our method outperforms the approach used by Zhang *et al*.^24^ when the same PRCA dataset is used.

## Discussion and Conclusion

Our approach can identify cancer-related miRNAs that may affect other molecules in the biological network based on three distinct features. For most of the analyzed cancer types, the integration of the three features led to the best performance in the identification of cancer-related miRNAs. However, the identification of BRCA-related miRNAs was improved when only two features, F1 and F3, were integrated, because F2 showed less power. Although the influence of these features can be controlled by assigning different weights to each feature, this requires prior knowledge, and therefore, the ability of generalization decreases. Here, we demonstrated that our order statistics-based method outperformed the average ranking ratio approach. To further investigate the influence of different weighting of features, we determined performance of feature integration by assigning different weights to each feature. These three features were integrated with various weights ranging from 0.1 to 1.0, resulting in a total of 1,000 test cases for each cancer type. We then selected the weight combinations providing the best performance, and the best combinations yielded performances similar to our approach for GBM, OVC and PRCA when selecting 100 candidate miRNAs, although our approach was outperformed in other cases. However, the best weight combinations were differed between different cancer types and the number of miRNAs selected as candidate cancer-related miRNA, confirming that it is hard to generalize optimal feature weights. Additionally, our order statistics approach significantly outperformed the worst weight combination, showing that our approach that considers ascending and descending rankings of the features can be generalized to any cancer type. Performances with various feature weights are described in Supplementary Table 18.

For GBM and OVC, we investigated top 20 miRNAs in detail to understand their roles in the cancer development, because more highly ranked miRNAs are more likely to be related to cancer. Functional roles of the top 20 candidate GBM miRNAs are described in Table 1 and Supplementary Table 19. Among them, miR-9/9* (ranked second and seventh), miR-181a/a* (ranked third and 10^th^), miR-21 (ranked fourth), miR-93 (ranked fifth), miR-34a (ranked sixth), miR-222 (ranked 13^th^), miR-17-5p/3p (ranked 14^th^ and 18^th^), and miR-181d (ranked 16^th^) were reported as important GBM-related miRNAs. MiR-9/9* function as onco-miRNAs or tumor suppressors, depending on the cellular environment. Schraivogel *et al*.^73^ reported that miR-9/9* are highly abundant in GBM stem cells and function as oncogenes by repressing tumor suppressor CAMTA1. Wu *et al*.^57^ reported that miR-9 is upregulated in glioma patients with high WHO grade (III-IV) and represents a useful prognostic factor for overall survival. Although these studies indicated that high miR-9/9* expression promotes tumor progression, Gomez *et al*.^74^ showed that, in GBM driven by EGFR mutation (EGFRvIII), miR-9 suppression leads to the enhanced tumor growth, because this miRNA targets FOXP1 transcription factor, and its upregulation can be oncogenic. MiR-181a/a* act as tumor suppressors, and Shi *et al*.^46^ showed that miR-181a inhibits cell growth and invasion and induces apoptosis in glioma cells. MiR-21 was reported to be an important biomarker in GBM. Chan *et al*.^75^ showed that miR-21 functions as an antiapoptotic factor by repressing apoptosis-related genes in GBM cells. Gabriely *et al*.^76^ showed that miR-21 promotes the activity of matrix metalloproteinases (MMPs) by targeting MMP inhibitors, which results in the increase of tumor invasiveness. Zhou *et al*.^52^ showed that miR-21 is involved in the regulation of EGFR and AKT pathways, and the suppression of cell growth in GBM. Furthermore, it was demonstrated^77^ that miR-93 targets integrin *β*8, affecting integrin *β*8-induced cell death in GBM. MiR-34a has been reported as an important regulator in GBM, and several studies^56^,^78^,^79^,^80^ reported that miR-34a functions as a tumor suppressor. MiR-34a targets several genes, such as c-Met, Notch-1, Notch-2, and CDK6, and regulates GBM-related pathways, e.g., p53 pathway. MiR-222 was shown^50^ to inhibit cell apoptosis and induce cell survival through the direct targeting of p53-upregulated modulator of apoptosis (PUMA) in GBM. MiR-222 was shown to be involved in tumorigenesis^81^ through the regulation of protein tyrosine phosphatase u (PTPu) expression in glioma cells. MiR-17-5p/3p were reported to regulate E2F1, PTEN, and MDM2. Srinivasan *et al*.^13^ demonstrated that the expression of miR-17 is associated with survival time in GBM. Additionally, decreased expression level of E2F1 and cyclin D1, which represent miR-17 targets, were shown to correlate with longer patient survival. MiR-17-5p was shown to target PTEN and miR-17-3p targets MDM2 51. MiR-17 overexpression was shown to increase the overall survival time. MiR-181d functions as tumor suppressor in GBM, and it may represent both a predictive biomarker in temozolomide therapy and a prognostic marker regulating MGMT expression in GBM^82^. Furthermore, Wang *et al*.^83^ showed that miR-181d functions as a tumor suppressor by regulating K-ras and Bcl-2.

Additionally, several studies^49^,^58^,^84^,^85^,^86^,^87^ showed that miR-22 (ranked first), miR-29a (ranked eighth), miR-30a-5p (ranked ninth), miR-30c (ranked 12^th^), miR-106a (ranked 15^th^), and miR-15b (ranked 17^th^) are primarily associated with cell apoptosis and proliferation in GBM. MiR-125b (ranked 11^th^) was shown to have different functions depending on the environment, and targets E2F1 and inhibits the proliferation of CD133-positive glioma stem cells^88^. However, it was shown that miR-125b function as oncogene as well, promoting proliferation and inhibiting the apoptosis of GBM stem cells^89^. Let-7a was ranked 20^th^ in our analysis, and Wang *et al*.^90^ showed that it directly targets K-ras and reduces glioma cell malignancy. Although let-7f (ranked 19^th^) was not described as GBM-related miRNA in HMDD, Yan *et al*.^91^ showed that it is involved in the inhibition of proliferation, migration, and invasion of glioma cells.

Functional roles of the top 20 OVC candidate miRNAs in microarray dataset are described in Supplementary Table 20. Among them, miR-21 (ranked third and 14^th^), miR-93 (ranked sixth), miR-20a (ranked 11^th^), miR-125b (ranked 13^th^), miR-16 (ranked 18^th^), and miR-27a (ranked 19^th^) are well-known OVC-related miRNAs. MiR-21 functions as an oncogene, and it is involved in tumorigenesis and tumor progression through the regulation of PTEN expression, an OVC-related tumor suppressor^70^. The suppression of miR-21 induces apoptosis of cisplatin-resistant OVC cells and the overexpression of miR-21 leads to the lowering of patient survival rate^67^. Similarly, miR-93 plays a key role in cell survival, since its suppression induces the apoptosis of OVC cells and it is involved in the determination of cisplatin chemosensitivity through the regulation of PTEN expression^92^. OVC cell survival was shown to be increased following miR-93 cell transfection^77^. Fan *et al*.^93^ showed that the overexpression of miR-20a promotes proliferation and invasion through direct targeting of amyloid precursor protein (APP) in OVC cells. MiR-125b was reported to be a tumor suppressor, since it suppresses cell proliferation through the regulation of BCL3, a proto-oncogene^94^, while it also inhibits tumor angiogenesis by regulating HER2 and HER3 95. Bhattacharya *et al*.^96^ showed that miR-16 regulates BMI-1 expression and that the downregulation of BMI-1 leads to the inhibition of proliferation and clonal growth of OVC cells. MiR-27a functions as an oncogene, and it controls the expression of multi-drug resistance (MDR)1/P-glycoprotein by targeting homeodomain interacting protein kinase (HIPK)2 that acts as a tumor suppressor^97^. Multiple studies^98^,^99^,^100^,^101^ showed that let-7b (ranked first), miR-29a (ranked fourth), miR-30c/30e* (ranked second and 17^th^) and miR-30b (ranked 20^th^) expression levels significantly differ between OVC tissues and controls. Furthermore, let-7b and miR-30c/30e* expression changes were associated with survival time, and the copy number of the mir-30b gene was shown to be increased in OVC^102^. MiR-29b/29c (ranked fifth and 16^th^) are involved in the development of cisplatin resistance^103^. Li *et al*.^104^ showed that miR-22 (ranked seventh) inhibits cell migration and invasion, and plays a key role in OVC metastasis. MiR-17 (ranked eighth in our analysis) is involved in the regulation of OVC-related pathways, suppressing LKB1-p53-p21/WAF1 pathway, which results in the induction of OVC stem cell development^69^. Yang *et al*.^105^ showed that miR-130a (ranked 10^th^) is involved in drug resistance mediated by DR1/P-glycoprotein in OVC cells.

Although miR-181a-2* (ranked ninth), miR-142-3p (ranked 12^th^), miR-24 (ranked 15^th^), and miR-29c (ranked 16^th^) are not indicated as OVC-related miRNAs in HMDD, Parikh *et al*.^106^ showed that miR-181a plays a crucial role in OVC progression, by promoting TGF-*β*-mediated epithelial-to-mesenchymal transition through the suppression of Smad7. Furthermore, the expression of miR-142-3p was shown to be highly correlated with a set of genes, including some cancer-related genes, and a set of methylation sites in OVC 107. MiR-24 acts as a tumor suppressor, inducing apoptosis in OVC cells^108^, while miR-29c was described previously^103^, together with miR-29b.

Functional roles of the top 20 OVC candidate miRNAs in RNA-Seq dataset are described in Supplementary Table 21. RNA-Seq and microarray datasets contain the expression levels of pre-miRNAs and mature miRNAs, respectively, however, eight common miRNAs in top 20 miRNAs can be observed: mir-181a-2/1 (ranked first and 13^th^), mir-22 (ranked third), mir-93 (ranked fourth), let-7b (ranked ninth), mir-125b-1 (ranked 11^th^), mir-30c-2 (ranked 14^th^), mir-30e (ranked 18^th^) and mir-17 (ranked 20^th^).

Let-7a (ranked fifth, sixth, and eighth), mir-200c (ranked 10^th^), and mir-25 (ranked 17^th^) are known as important OVC-related miRNAs. Let-7a-2/3/1 were reported as OVC prognosis markers. The methylation of let-7a-3 was identified in epithelial OVCs, and it affects the expressions of insulin-like growth factor 2 (IGF2) and patient survival^109^. Low let-7a expression and high LIN28B expression were correlated with poorer prognosis^110^. Two studies^111^,^112^ showed that miR-200c acts as a regulator, reducing tumor burden and increasing the sensitivity to chemotherapy by regulating TUBB3 (class III *β*-tubulin gene). High miR-200c expression suppresses TUBB3 expression, which ultimately prolongs survival. Zhang *et al*.^113^ showed that the overexpression of miR-25 induces cell proliferation by directly repressing the expressions of a pro-apoptotic protein Bim in OVC. Two studies^114^,^115^ showed that miR-92a directly represses the expression of integrin *α*5, and it was shown that the increased expression of this integrin leads to the poorer survival of patients with stage III OVC. Moreover, Cai *et al*.^116^ showed that let-7e (ranked seventh) is involved in the development of cisplatin resistance in OVC, while miR-30a/99b (ranked 12^th^ and 15^th^, respectively), were shown to be differentially expressed between OVC tissues and controls^99^,^100^. Additionally, the functional roles of the top 20 PRCA and BRCA candidate miRNAs are described in Supplementary Tables 22 and 23.

We demonstrated here that our proposed approach outperforms the approaches based on the datasets showing differentially expressed miRNAs in the identification of cancer-related miRNAs. Therefore, our method can be widely applied to other cancer datasets and may contribute to the elucidation of cancer-related miRNA mechanisms. Furthermore, providing the ranking information, which allows the selection of a small number of highly relevant miRNAs, represent an advantage of our method.

## Additional Information

**How to cite this article**: Jin, D. and Lee, H. Prioritizing cancer-related microRNAs by integrating microRNA and mRNA datasets. *Sci. Rep.* **6**, 35350; doi: 10.1038/srep35350 (2016).

## Supplementary Material

Supplementary Tables

Supplementary Table 1

Supplementary Table 2

Supplementary Table 3

Supplementary Table 4

Supplementary Table 5

Supplementary Table 6

Supplementary Table 7

Supplementary Table 8

Supplementary Table 9

Supplementary Table 10

Supplementary Table 11

Supplementary Table 12

Supplementary Table 13

Supplementary Table 14

Supplementary Table 15

Supplementary Table 16

Supplementary Table 17

Supplementary Table 18

Supplementary Table 19

Supplementary Table 20

Supplementary Table 21

Supplementary Table 22

Supplementary Table 23

## Figures and Tables

**Table 1:** Studies on top 20 miRNAs identified in GBM samples.

Rank	miRNA	HMDD	Evidence	Reference
1	miR-22	O	Correlation between miRNA expression changes and alteration in mRNA levels of different glioma cells after PUFA or temozolomide treatment	Faragó *et al*.^84^
2,7	miR-9*/9	O	CAMTA1 is a novel tumor suppressor regulated by miR-9/9* in glioblastoma stem cells	Schraivogel *et al*.^73^
3,10	miR-181a/181a*	O	Hsa-mir-181a and hsa-mir-181b function as tumor suppressors in human glioma cells	Shi *et al*.^46^
4	miR-21	O	MicroRNA-21 is an antiapoptotic factor in human glioblastoma cells	Chan *et al*.^75^
5	miR-93	O	MicroRNA miR-93 promotes tumor growth and angiogenesis by targeting integrin-*β*8	Fang *et al*.^77^
6	miR-34a	O	MicroRNA-34a inhibits glioblastoma growth by targeting multiple oncogenes.	Li *et al*.^78^
8	miR-29a	O	Over-expression of miR-29a/29b promotes apoptosis of GICs (GBM initiating cells) by inhibiting MCL1 protein expression	Aldaz, *et al*.^85^
9	miR-30a-5p	O	MiR-30a-5p antisense oligonucleotide suppresses glioma cell growth by targeting SEPT7	Jia *et al*.^49^
11	miR-125b	O	MiR-125b regulates the proliferation of glioblastoma stem cells by targeting E2F2	Wu *et al*.^88^
12	miR-30c	O	Effect of miR-21 and miR-30b/c on TRAIL-induced apoptosis in glioma cells	Quintavalle *et al*.^86^
13	miR-222	O	MiR-221 and miR-222 target PUMA to induce cell survival in glioblastoma	Zhang *et al*.^50^
14,18	miR-17-5p/3p	O	Stress response of glioblastoma cells mediated by miR-17-5p targeting PTEN and the passenger strand miR-17-3p targeting MDM2.	Li *et al*.^51^
15	miR-106a	O	MiR-106a inhibits glioma cell growth by targeting E2F1 independent of p53 status	Yang *et al*.^58^
16	miR-181d	O	MiR-181d acts as a tumor suppressor in glioma by targeting K-ras and Bcl-2	Wang *et al*.^83^
17	miR-15b	O	MiR-15b and miR-152 reduce glioma cell invasion and angiogenesis via NRP-2 and MMP-3	Zheng *et al*.^87^
19	let-7f	X	Let-7f Inhibits glioma cell proliferation, migration, and invasion by targeting periostin	Yan *et al*.^91^
20	let-7a	O	Overexpressed let-7a inhibits glioma cell malignancy by directly targeting K-ras, independently of PTEN	Wang *et al*.^90^

Columns 1 and 2 present the ranking and name of miRNAs, respectively. In column 3, GBM-related miRNA in HMDD are marked. Columns 4 and 5 summarize representative evidence and studies supporting the role of miRNAs in GBM, respectively.
