# Supplementary material for: Prioritizing cancer-related microRNAs by integrating microRNA and mRNA datasets
Source: Sci Rep. 2016 Oct 13;6:35350. doi: 10.1038/srep35350 (PMC5062133; doi:10.1038/srep35350)
Supplement: Supplementary Tables [file srep35350-s1.pdf]

Supplementary data:
Prioritizing cancer-related microRNAs by integrating microRNA and mRNA datasets

Daeyong Jin and Hyunju Lee*

Supplementary Table List (Excel File)

Table	Description
1	Feature integration process for several cases.
2	The comparison of proposed integration method with other methods.
3	miRNA rankings, integrated scores and cumulative ratios of cancer-related miRNAs for GBM.
4	miRNA rankings, integrated scores and cumulative ratios of cancer-related miRNAs for OVC (Microarray).
5	miRNA rankings, integrated scores and cumulative ratios of cancer-related miRNAs for OVC (RNA-Seq).
6	miRNA rankings, integrated scores and cumulative ratios of cancer-related miRNAs for PRCA.
7	miRNA rankings, integrated scores and cumulative ratios of cancer-related miRNAs for BRCA.
8	Gene-miRNA interactions related to GBM genes.
9	Gene-miRNA interactions related to OVC genes.
10	Reference GBM related pathways.
11	Reference OVC related pathways.
12	KEGG pathways related to top20 GBM candidate miRNAs.
13	KEGG pathways related to top20 OVC candidate miRNAs.
14	GO biological functions related to top20 GBM candidate miRNAs.
15	GO biological functions related to top20 OVC candidate miRNAs.
16	Survival analysis for top100 GBM candidate miRNAs.
17	Survival analysis for top100 OVC candidate miRNAs.
18	Performance for identifying cancer miRNAs on different weights across the cancers.
19	Functional role of the top 20 GBM candidate miRNAs.
20	Functional role of the top 20 OVC (microarray) candidate miRNAs.
21	Functional role of the top 20 OVC (RNA-Seq) candidate miRNAs.
22	Functional role of the top 20 PRCA candidate miRNAs.
23	Functional role of the top 20 BRCA candidate miRNAs.
